# Complete Genome Sequence of *Campylobacter concisus* ATCC 33237^T^ and Draft Genome Sequences for an Additional Eight Well-Characterized *C. concisus* Strains

**DOI:** 10.1128/genomeA.00711-17

**Published:** 2017-07-20

**Authors:** Angela J. Cornelius, William G. Miller, Albert J. Lastovica, Stephen L. W. On, Nigel P. French, Olivier Vandenberg, Patrick J. Biggs

**Affiliations:** a Institute of Environmental Science and Research Ltd. (ESR), Christchurch, New Zealand; b U.S. Department of Agriculture, Agricultural Research Service, Albany, California, USA; c University of Western Cape, Bellville, South Africa; d Lincoln University, Lincoln, New Zealand; e Massey University, Palmerston North, New Zealand; f National Reference Centre for Campylobacter, Laboratoire Hospitalier Universitaire de Bruxelles (LHUB-ULB), Brussels, Belgium; g School of Public Health, Université Libre de Bruxelles, Brussels, Belgium; h New Zealand Genomics Limited, Palmerston North, New Zealand

## Abstract

We report the complete genome sequence of the *Campylobacter concisus* type strain ATCC 33237 and the draft genome sequences of eight additional well-characterized *C. concisus* strains. *C. concisus* has been shown to be a genetically heterogeneous species, and these nine genomes provide valuable information regarding the diversity within this taxon.

## GENOME ANNOUNCEMENT

The cells of *Campylobacter concisus* are Gram-negative, non-spore-forming (1), small (0.5 × 4 µm), and curved with rounded ends (2). *C. concisus* has been isolated from a variety of sites from the human body, including the gingival crevices of patients with gingivitis and periodontitis, stomach and esophagus biopsy specimens, blood, and both normal and diarrheic stools (2). In South Africa, *C. concisus* is the second most commonly isolated *Campylobacter* species in pediatric diarrheic stools (3). This species has also been shown to be phenotypically (4, 5) and genetically (6–11) heterogeneous.

Nine strains were sequenced in this study. *C. concisus* ATCC 33237 is the type strain of this species and was sequenced to completion. One strain, CCUG 19995, was isolated in 1987 in Sweden from a patient with pyrexia and exanthema. The remaining seven strains (Lasto28.99, Lasto61.99, Lasto64.99, Lasto127.99, Lasto205.94, Lasto220.96, and Lasto393.96) were isolated in South Africa between 1994 and 1999 from patients with dysentery, diarrhea, or loose mucoid stools. Strains CCUG 19995, Lasto127.99, and Lasto393.96 are from genomospecies 2, 5, and 6 (12, 13), respectively, while the remaining six strains are members of genomospecies 1 (12). The draft genomes of the eight strains have been well characterized and are genetically diverse (12).

Sequencing of ATCC 33237^T^ was undertaken using the 454 FLX+ (Titanium chemistry), Illumina (HiSeq), and PacBio platforms. The 454 and Illumina reads were assembled using Newbler (version 2.6) (14, 15) into a single scaffold that was closed using PCR amplification and Sanger sequencing. PacBio sequencing was performed to address repeat regions within the genome and an optical bacterial restriction map (16, 17) (restriction enzyme *Spe*I; OpGen, Gaithersburg, MD) was used to validate the assembly. Protein-coding genes, ribosomal loci, tRNAs, and gene start points were identified as described (18). Annotation was performed by BLASTP comparison to the proteomes of completed *Campylobacter* genomes or to proteins in the NCBI nonredundant database, and by identification of Pfam domains (v.27.0) (19).

Sequencing of CCUG 19995, Lasto28.99, Lasto61.99, Lasto64.99, Lasto127.99, Lasto205.94, Lasto220.96, and Lasto393.96 was undertaken using an Illumina MiSeq. Average coverage between 152× and 254× was achieved. Velvet (version 1.2.10) (20) was used to assemble the short reads, which were quality trimmed using SolexaQA++ (21) at a quality threshold of 0.01, and then sorted by length to remove all resulting reads less than 50 bases long. The draft genomes were annotated using the Prokaryotic Genome Annotation Pipeline (22). The *N*_50_ values for these genomes, as calculated using the QUAST (23) online calculator (http://quast.bioinf.spbau.ru/), were between 134,605 and 349,534 bp.

The two genomes from genomospecies 2 and 5 (CCUG 19995 and Lasto127.99) had G+C contents of 39.4%, compared to G+C values of between 37.4% and 37.7% for the seven genomes from genomospecies 1 and 6.

### Accession number(s).

The genome sequences of ATCC 33237^T^, CCUG 19995, Lasto28.99, Lasto61.99, Lasto64.99, Lasto127.99, Lasto205.94, Lasto220.96, and Lasto393.96 have been deposited at GenBank under the accession numbers CP012541, NDYN00000000, NDYO00000000, NEFM00000000, NDYP00000000, NDYQ00000000, NDYR00000000, NDYS00000000, and NDYT00000000, respectively. The versions described in this paper are the first versions.
